# Post treatment quality of life among Sri Lankan women with breast cancer

**DOI:** 10.1186/s12885-021-08055-5

**Published:** 2021-03-23

**Authors:** Ravindri Jayasinghe, Ashan Fernando, Umesh Jayarajah, Sanjeewa Seneviratne

**Affiliations:** Department of Surgery, Faculty of Medicine, University of Colombo, P.O. Box 271, Kynsey Road, Colombo 8, Western Province Sri Lanka

**Keywords:** Quality of life, Breast cancer, Mastectomy, Breast-conserving surgery, Sri Lanka

## Abstract

**Background:**

Breast cancer and its treatment imposes a significant effect in the quality of life (QOL) of women. Being a developing country with contrasting social and cultural norms to the West, Sri Lankan women may have a different experience on QOL following surgical treatment of breast cancer. This study was conducted to evaluate post-treatment QOL in breast cancer patients and to determine its association with the type of surgery.

**Methods:**

A cross sectional study was carried out. Fifty four women with non-metastatic breast cancer who underwent surgery for breast cancer at the Professorial Surgical Unit, Colombo during 2015–2018 and completed a minimum of one year follow up after surgery were invited to participate. Fifty-four women who responded were assessed using the validated EORTC QLQ-C30 and QLQ-BR23 questionnaires. Non-parametric tests were used for statistical analyses.

**Results:**

The mean age was 59 years (range 36–81). A majority (61%, *n* = 35) underwent mastectomy and the rest (*n* = 19, 45%) breast conservation surgery (BCS). The mean QLQ-C30 score was 68.8 (range 8.3–100) and the mean scores for physical function, role function, emotional function, cognitive function, and social function were 71.4, 81.5, 77.0, 80.2, and 86.4, respectively. The mean scores for body image, sexual functioning, sexual enjoyment, future perspective, systemic therapy, breast symptoms, arm symptoms, and hair loss assessed by the QLQ-BR23 were 76.4, 18.3, 33.3, 73.6, 30.5, 16.2, 23.4 and 32.7, respectively. No significant differences (*P* > 0.05) were noted in global health status, physical function, role function, emotional function, cognitive function and social function between BCS and mastectomy. QLQ-BR23 body image, sexual functioning, sexual enjoyment and future perspective also did not differ significantly (*p* > 0.05) between the two groups.

**Conclusions:**

Sexual functioning and enjoyment, breast and arm symptoms and hair loss contributed to poor QOL while the impact on global health status including physical, social and emotional functions were minimal. Type of surgery did not appear to be associated with QOL. Future studies with a larger sample sizes will be helpful to further study these factors.

**Supplementary Information:**

The online version contains supplementary material available at 10.1186/s12885-021-08055-5.

## Background

Breast cancer is the most commonly diagnosed cancer in women with over two million new patients diagnosed in 2018. Furthermore, it is the commonest cause for cancer-related death in women with over 600,000 deaths the same year [1]. In Sri Lanka, breast cancer is the commonest malignancy with an increasing incidence, especially in the post-menopausal women [2, 3].

Advancements in diagnosis and treatment have substantially improved the survival rates from breast cancer over last two to three decades with a majority of women achieving a complete cure [4, 5]. Although these advances would add ‘years to patients’ lives’, it may not add ‘life to patients’ years’ shifting the focus towards improving the quality of life of patients [6]. This has led to post-treatment QOL being recognized as an important issue among the survivors due to the negative impacts of treatment [6, 7]. QOL in these women is affected not only due to the morbidity linked with the treatment but also due to the additional burden from psychosocial aspects such as changes in lifestyle, sexual dysfunction, and alteration of body image [6].

In the South East Asian region, the evaluation of response to therapy is largely limited to the use of traditional markers such as disease free survival and overall survival. However, worldwide, especially in the West there is an increasing focus towards optimising the health-related quality of life among survivors [8]. The concept of QOL is broad given the complexity of the aspects involved. Health-related quality of life (HRQL) broadly involves the patients’ outlook on the impact of illness, impairments, and the impact of therapeutic interventions in the context of quality of life [9]. There are a range of generic and specific instruments developed to assess the quality of life (QOL). As generic instruments are not specific to a particular health condition, specific instruments are made with improved sensitivity to detect changes after an intervention. Amongst the specific instruments available, the instrument developed by the European organization for Research and Treatment of Cancer (EORTC) to evaluate the health-related quality of life by a core questionnaire QLQ-C30 stands out being widely used in literature with proven reliability and reproducibility [10, 11]. The validity of this tool in evaluating health-related quality of life among Sri Lankan women with breast cancer had been proven previously [12].

Two main modes of surgical treatment of breast cancer include breast conservation surgery (BCS) and mastectomy. Evidence has proven BCS followed by whole breast irradiation and total mastectomy to carry nearly equal oncological outcomes in early breast cancer [13]. However, the choice on type of surgery would depend on several patient and disease related factors. Disease related factors such as inflammatory breast cancer, multicentric disease, prior history of radiation to the chest wall, large tumour size in relation to the breast and patient related factors like pregnancy are contraindications for BCS. Furthermore, patients may opt for mastectomy due to several reasons such as fear of recurrence, aversion to radiotherapy and the surgeons’ expertise. Breast cancer surgery in most patients would contribute to some degree of both short and longer-term morbidity which may vary depending on patient and tumour factors. A systematic review in 2016 established the negative implications of surgical complications on patient psychosocial outcomes and the QOL [14].

Several studies over the years have focused on QOL after surgery for breast cancer. Although most have shown BCS to be superior to mastectomy, some had shown no significant difference in QOL [15–18]. Several long term follow-up studies have shown no improvement in body image, sexual function over time after BCS versus mastectomy [15, 19]. Most literature evaluating post-surgical QOL in patients with breast cancer are from the Western world which might significantly differ from the South Asian region due to the sociocultural differences. Even amongst Sri Lankan women, differences are likely to exist depending on the different social, ethnic, and educational groups. Therefore, there is a gap in knowledge in the local context regarding many aspects of QOL after treatment of breast cancer. This study was conducted with the aim of evaluating the post-treatment quality of life (QOL) in breast cancer patients and to determine its association with the type of surgery.

## Methods

A cross sectional study was carried out. Fifty four women with non-metastatic breast cancer who underwent surgery for breast cancer at the Professorial Surgical Unit, Colombo during 2015–2018 and completed a minimum of one year follow up after surgery having an ECOG score of 0–2 were invited via phone calls and mail to participate in the study. The patients were recruited in the immediate postoperative period and followed up. Only the ones with a follow-up duration of minimum one year were included in the study. Fifty-four women who responded were assessed using the validated EORTC QLQ-C30 and QLQ-BR23 questionnaires. Validated Sinhala, Tamil and English versions of the questionnaires were used [20, 21]. Non-parametric tests were used for statistical analyses.

Women with a previous history of breast or other types of cancer were excluded. Ethical approval for this study was obtained from the Ethical Review Committee, Faculty of Medicine Colombo (EC: 17–126).

The QLQ-C30 questionnaire includes 30 items to assess five functional scales (physical, cognitive, role, emotional, and social), three symptom scales (Fatigue, pain, nausea and vomiting) and a global health and quality of life scale [10]. QLQ-BR23 includes 23 items including four functional scales and four symptom scales where a higher score in functional scales would represent better functioning and a higher score in symptom scales would indicate increased symptom related issues. Additional data were obtained with regard to the type of surgery - BCS or mastectomy and the menopausal status.

### Statistical analysis

The data were coded and analysed according to the scoring protocol described in the EORTC QLQ-30 manual by Fayers et al. using SPSS version 24 [10, 22]. Non-parametric tests including Chi square (*χ*^2^) test, Mann-Whitney U test and Spearman correlation test were used for univariate statistical analyses. QOL scores were compared between women undergoing BCS versus mastectomy. A *p* value < 0.05 was considered as statistically significant.

## Results

Fifty-four female patients (16.8%) from a total of 320 eligible women who had undergone surgery for non-metastatic breast cancer and consented to participate were included in the study. The mean age was 59.0 years (range 36–81) with 61.1% (*n* = 33) being less than 60 years. Majority of the women were postmenopausal (85.2%%, *n* = 46). A majority (61.1%, *n* = 35) underwent mastectomy as the primary surgery and the rest (45%, *n* = 19) underwent BCS. None of the women who had undergone mastectomy and included in the study had breast reconstruction. Axillary node dissection was performed in 59.2% (*n* = 32) and the rest only a sentinel lymph node biopsy. The patients had a median follow-up duration of > 2 years (Table 1).

**Table 1:** Demographic and clinical characteristic of study participants

Variables	Frequency	Percentage
*Age*		
≤ 60 years	33	61.11%
> 60 years	21	38.9%
*Marital status*		
Married	51	94.4%
Unmarried	3	5.6%
*Educational level*		
Primary	4	7.4%
Secondary	43	79.6%
Graduate	2	3.7%
Postgraduate	3	5.6%
*Occupation*		
Actively employed	10	18.5%
Retired/homemaker	5	9.3%
Not available	39	72.22%
*Menopausal status*		
Premenopausal	7	13.0%
Postmenopausal	46	85.2%,
*Tumour size*		
T1	15	30.6%
T2	27	55.1%
T3	5	10.2%
T4	2	4.1%
*Node positivity*		
N0	17	37.0%
N1	13	28.3%
N2	11	23.9%
N3	5	10.9%
*Metastasis*		
M0	27	49.1%
Mx	12	21.8%
*Type of surgery*		
Breast conservation surgery	19	35.2%
Mastectomy	33	61.1%
*Time elapsed following surgery*		
> 2 years	38	70.38%
2 years	15	27.8%
*Axillary surgery*		
Sentinel lymph node biopsy	16	29.6%
Axillary lymph node dissection	32	59.3%
*Adjuvant chemotherapy*		
Yes	23	42.6%
No	9	16.7%
No information	22	40.7%
*Adjuvant radiotherapy*		
Yes	29	53.7%
No	3	5.6%
No information	22	40.7%
*Hormone Therapy*		
Yes	20	37%
No	12	22.2%
No information	22	40.7%

The mean EORTC QLQ-C30 score was 68.8 (range: 8.3–100) and the mean scores for physical function, role function, emotional function, cognitive function, and social function were 71.4 (range: 33.3–100), 81.5 (range: 0–100), 77 (range: 0–100), 80.2 (range: 33.3–100), 86.4 (range: 0–100), respectively. The EORTC QLQ-C30 scores in the less than 60-year age group (*n* = 33) was not any different than the older patients (greater than or equal to 60 years) (Table 2).

**Table 2:** Analysis of factors associated with quality of life

Variables		***Age***		Type of surgery		Axillary surgery	
		≤60 years	> 60 years	BCS	Mastectomy	Sentinel lymph node biopsy	Axillary lymph node dissection
EORTC QLQ-C30* score							
**Global health status**	Median	83.3	58.3	83.3	66.7	70.8	75
	Range	8.3–100	25–100	25–100	8.33–100	8.33–100	25–100
	p	0.135		0.414		0.550	
**Physical function**	Median	73.33	73.33	73.33	73.33	66.67	80
	Range	33.3–100	41.7–100	33.3–100	33.3–100	33.3–100	33.3–100
	p	0.531		0.962		0.181	
**Role function**	Median	83.3	83.3	83.3	83.3	91.7	83.3
	Range	0–100	16.7–100	16.7–100	0–100	66.7–100	16.7–100
	p	0.087		0.429		0.78	
**Emotional function**	Median	91.7	75	91.7	75	91.7	91.7
	Range	0–100	16.7–100	0–100	0–100	16.7–100	16.7–100
	p	0.587		0.745		0.759	
**Cognitive function**	Median	83.3	83.3	83.3	83.3	83.3	83.3
	Range	33.3–100	50–100	50–100	33.3–100	33.3–100	50–100
	p	0.108		0.309		0.501	
**Social function**	Median	100	100	100	100	100	100
	Range	16.7–100	0–100	0–100	16.7–100	50–100	0–100
	p	0.27		0.193		0.81	
BR 23 Score**							
**Body image**	Median	86.11	83.33	100	83.33	95.83	83.33
	Range	16.7–100	16.7–100	16.7–100	41.7–100	16.7–100	16.7–100
	p	0.874		0.375		0.645	
**Sexual functioning**	Median	16.7	0	16.7	8.3	16.7	0
	Range	0–100	0–33.3	0–100	0–100	0–100	0–100
	p	0.145		0.172		0.236	
**Sexual enjoyment**	Median	33.3	16.7	33.3	33.3	33.3	33.3
	Range	0–100	0–66.7	0–100	0–100	0–100	0–100
	p	0.57		0.283		0.393	
**Future perspective**	Median	100	66.7	100	100	83.3	100
	Range	0–100	0–100	0–100	0–100	0–100	0–100
	p	0.227		0.949		0.595	
**Systemic therapy**	Median	23.8	28.5	28.5	23.8	35.7	19.1
	Range	0–90.4	0–90.4	0–85.7	0–90.4	9.5–90.4	0–90.5
	p	0.636		0.811		0.009	
**Breast symptoms**	Median	16.7	16.7	16.7	16.7	16.7	16.7
	Range	0–66.7	0–66.7	0–66.7	0–33.3	0–66.7	0–66.7
	p	0.27		0.469		0.393	
**Arm symptoms**	Median	22.2	22.2	11.1	22.2	22.2	22.2
	Range	0–77.8	0–66.7	0–55.6	0–77.8	0–66.7	0–77.8
	p	0.689		0.185		0.84	
**Hair loss**	Median	0	33.3	16.7	0	66.7	0
	Range	0–100	0–100	0–100	0–100	0–100	0–100
	p	0.084		0.859		0.009	

* European organization for Research and Treatment of Cancer Quality of Life Questionnaire C30

** European organization for Research and Treatment of Cancer Quality of Life Questionnaire BR 23

The mean scores for body image, sexual functioning, sexual enjoyment, future perspective, systemic therapy, breast symptoms, arm symptoms, and hair loss assessed by the QLQ BR-23 were 76.4 (range: 16.67–100), 18.3 (range: 0–100), 33.3 (range: 0–100), 73.6 (range: 0–100), 30.5 (range: 0–90.5), 16.2 (range: 0–66.7), 23.45 (range: 0–77.8), 32.7 (range: 0–100), respectively.

Significant negative correlation was noted between the overall EORTC QLQ-C30 score and the decreasing age (*p* = 0.022). No significant difference was noted in the global health status (*p* = 0.41), physical function (*p* = 0.96), role function (*p* = 0.42), emotional function (*p* = 0.74), cognitive function (*p* = 0.30), and social function (*p* = 0.19) parameters in the two groups who underwent BCS versus mastectomy as reported by the EORTC QLQ-C30. The parameters assessed by the QLQ BR-23 such as body image (*p* = 0.37), sexual functioning (*p* = 0.17), sexual enjoyment (*p* = 0.28), future perspective (*p* = 0.94), systemic therapy (*p* = 0.81), breast symptoms (*p* = 0.46), arm symptoms (*p* = 0.18), and hair loss (*p* = 0.859) were also similar in the two groups who underwent BCS compared with mastectomy (Table 2).

## Discussion

This cross-sectional study evaluated the post-treatment QOL in Sri Lankan female patients diagnosed with breast cancer using the validated EORTC QLQ-C30 and BR-23 tools have shown substantially poor QOL in sexual functioning and enjoyment, breast and arm symptoms and hair loss domains while the impact on global health status including physical, social and emotional functions were minimal. The mean EORTC QLQ-C30 score of 68.8 indicated that our patients health related quality of life (HRQL) is comparable with average global figures (mean 61.8+/− 24.6) as well as previously published local figures (mean 50 +/− 24.3) [12, 23]. However, our study did not demonstrate an association with the type of surgery and the post-treatment QOL of patients according to EORTC QLQ-C30 and QLQ-BR23 scores (*P* > 0.05). In addition, a negative correlation was observed with the overall EORTC QLQ-C30 score and the age (*p* = 0.022) where younger patients showed a significantly better QOL scores. This finding is different to that of most studies from the west which could be because the value of the intact breast is perceived differently in younger women than older women as younger women are more sexually active than older women unlike in the western world [24]. This is evident in regional studies which is keeping in with our findings [25].

A majority of previous studies investigating QOL with type of breast surgery from Western countries have shown conflicting results although most studies have shown BCS to be superior to mastectomy [15–18, 26, 27]. A study by Chow et al. on symptom burden and QOL in breast cancer patients treated with BCS versus mastectomy showed mastectomy patients to have a significantly lower QOL, lower physical and emotional wellbeing, higher pain, anxiety, drowsiness and loss of appetite compared with BCS [26]. However, in that study, the QOL was assessed using a different tool compared to the present study [26]. A study done in Germany by Engel et al. using similar assessment tools concluded that the patients undergoing BCS had better scores in most variables and had a significantly better overall QOL. In this study, mastectomy patients had lower body image, limited role function, less sexual activity, increased insecurity and had their day today activities affected to a greater extent [19].

Few long-term follow-up studies have shown no differences in body image, sexual function over time among patients who underwent BCS in comparison with mastectomy [15, 27]. A large prospective cohort study by Ganz et al. comparing patients undergoing BCS versus mastectomy found no significant differences in quality of life, psychosocial adjustment, rehabilitation needs, or mood [15]. Another large prospective cohort study by Browne et al. that analysed the association between complications and QOL after breast reconstruction and mastectomy showed that surgical complications had little or no association with the quality of life among patients who underwent mastectomy with or without reconstruction [27]. Furthermore, surgical complications did not have a significant impact on the their physical wellbeing scores [27]. The reasons behind the unexpected lack of improvement in quality of life after BCS may be explained by the fear of recurrence or the effects of post-operative radiotherapy. This could be further influenced by local sociocultural and educational factors as well as personal beliefs. Furthermore, absence of a deterioration of body image in our sample may also suggest the absence of a difference in QOL parameters in relation to type of surgery.

In comparison with the global values for QLQ-30 in women after breast cancer treatment, the role function, emotional function, and social function showed substantially better QOL values. However, compared to global parameters, the scores of BR-23 such as body image, sexual functioning and sexual enjoyment appear to be lower [23]. The reasons for these differences could be due to the sociocultural disparities in the local population compared to the women from the Western countries. Most evidence on QOL after breast cancer surgery are from the Western world where the ‘value’ of an intact breast may vary from Sri Lankan women due to differences in socio-cultural values and body image. Most women are reluctant to bring out these sensitive issues related to sexual function and enjoyment in the local context. Higher mean age of the study population (59 years) with a majority of the women being postmenopausal (85.2%) might also have contributed to the observed lack of difference between mastectomy and BCS.

According to our findings, the low sores in breast related symptoms measured by BR-23 seems to be a major contributing factor for the lower QOL in breast cancer patients. Taking this into consideration, it is necessary to take measures to address the burden of breast related symptoms of these patients following surgery as these are easily preventable with adequate care. The HRQL of these patients may be improved by simple measures such as addressing sexual issues by referring them for counselling and prescribing topical applications, offering physiotherapy to alleviate arm symptoms, provision of wigs to combat hair loss following treatment. Furthermore, in the Sri Lankan context patients would be reluctant to bring out these sensitive issues themselves with the doctors. Provision of regular contact with the patients through trained cancer care nurses to recognize these issues and provide advice may help improve QOL in these domains which ultimately will help improve overall QOL. Sri Lanka is a country with a strong preventive health care system. With the increasing cancer burden, the healthcare policy makers should strengthen the public health system by incorporating cancer care nurses into the system and they can address the issues of these patients as they arise.

Our study was the first of its kind performed in the Sri Lankan context. The main limitations of this study is the smaller sample size which may not be representative, the inequality of age distribution and the absence of a control group. Majority of the sample being older women could also contribute to the QOL scores mainly due to the sociocultural factors coming into play in the local context. Several other contributors for morbidity following surgery for breast cancer such as sensory complaints of the ipsilateral arm, duration of disease, and a comparison before and after surgery was not considered in the present study which seem necessary in evaluating the QOL. Qualitative methods in future studies may provide further insight. Further prospective studies with larger sample sizes are deemed essential in the local context incorporating these other factors that may affect QOL among breast cancer patients.

## Conclusions

Sexual functioning and enjoyment, breast and arm symptoms and hair loss contributed to poor QOL in women after treatment for breast cancer while the impact on global health status including physical, social and emotional functions were minimal. Type of surgery did not appear to be associated with QOL. Age was the only statistically significant factor associated with QOL, where younger patients showed a significantly better QOL. Measures should be implemented to help women with breast related symptoms especially sexual, arm and hair loss related symptoms which contribute significantly to poor QOL following surgery. Further studies with larger sample sizes will be helpful to confirm these findings and to identify strategies to improve QOL in these specific domains.

## Supplementary Information

**Additional file 1.** STROBE Statement—Checklist of items that should be included in reports of cross-sectional studies.

## Data Availability

The data used in the above analysis will be available on reasonable request from the corresponding author.

## Abbreviations

QOL: Quality of life
BCS: Breast conservation surgery
HRQL: Health related quality of life
EORTC: European organization for Research and Treatment of Cancer

## References

[1] World Health Organization. International Agency for Research on Cancer. Cancer Today - GLOBOCAN. Accessed from: https://gco.iarc.fr/today/home. Accessed 03 Jan 2021.

[2] Fernando A, Jayarajah U, Prabashani S, Fernando EA, Seneviratne SA (2018). Incidence trends and patterns of breast cancer in Sri Lanka: an analysis of the national cancer database. BMC Cancer.

[3] Jayarajah U, Varothayan S, Jayasinghe R, Seneviratne S: Present status of cancer burden in Sri Lanka based on GLOBOCAN estimates. *South Asian J Cancer* 2021, 10:In Press.

[4] Kesson EM, Allardice GM, George WD, Burns HJ, Morrison DS (2012). Effects of multidisciplinary team working on breast cancer survival: retrospective, comparative, interventional cohort study of 13 722 women. BMJ.

[5] Basnayake O, Jayarajah U, Seneviratne S. Management of axilla in breast cancer: the past, present and the future. Sri Lanka Journal of Surgery. 2018:36(4).

[6] Bertan FC, EKd C. Qualidade de vida e câncer: revisão sistemática de artigos brasileiros. Psico (Porto Alegre). 2009:366–72.

[7] Nounou MI, ElAmrawy F, Ahmed N, Abdelraouf K, Goda S, Syed-Sha-Qhattal H: Breast Cancer: Conventional Diagnosis and Treatment Modalities and Recent Patents and Technologies. *Breast Cancer* 2015, 9s2:BCBCR.S29420. 10.4137/BCBCR.S29420 PMC4589089 26462242

[8] Aaronson N (1995). Quality of life and clinical trials. Lancet.

[9] Barbosa PA, Cesca RG, Pacífico TED, Leite ICG (2017). Quality of life in women with breast cancer, after surgical intervention, in a city in the zona da Mata region in Minas Gerais, Brazil. Revista Brasileira de Saúde Materno Infantil.

[10] Aaronson NK, Ahmedzai S, Bergman B, Bullinger M, Cull A, Duez NJ, Filiberti A, Flechtner H, Fleishman SB, de Haes JC (1993). The European Organization for Research and Treatment of Cancer QLQ-C30: a quality-of-life instrument for use in international clinical trials in oncology. JNCI.

[11] Fas M, Mrdo L, Ms M (2013). Validity, reliability and understanding of the eortc-c30 and eortcBr23, quality of life questionnaires specific for breast cancer. Rev Bras Epidemiol.

[12] Jayasekara H, Rajapaksa LC, Aaronson NK (2008). Quality of life in cancer patients in South Asia: psychometric properties of the Sinhala version of the EORTC QLQ-C30 in cancer patients with heterogeneous diagnoses. Qual Life Res.

[13] National Institutes of Health: Treatment of early stage breast cancer. In: *NIH Consensus Development Conference:* 1991; 1991.

[14] Pinto A, Faiz O, Davis R, Almoudaris A, Vincent C. Surgical complications and their impact on patients’ psychosocial well-being: a systematic review and meta-analysis. BMJ Open. 2016:6(2). 10.1136/bmjopen-2014-007224 PMC4762142 26883234

[15] Ganz PA, Anne Schag CC, Lee JJ, Polinsky ML, Tan SJ (1992). Breast conservation versus mastectomy. Is there a difference in psychological adjustment or quality of life in the year after surgery?. Cancer.

[16] Kiebert G, De Haes J, Van de Velde C (1991). The impact of breast-conserving treatment and mastectomy on the quality of life of early-stage breast cancer patients: a review. J Clin Oncol.

[17] Rietman JS, Dijkstra PU, Hoekstra HJ, Eisma WH, Szabo BG, Groothoff JW, Geertzen JH (2003). Late morbidity after treatment of breast cancer in relation to daily activities and quality of life: a systematic review. EJSO.

[18] Freitas-Silva R, Conde DM, Rd F-J, Martinez EZ (2010). Comparison of quality of life, satisfaction with surgery and shoulder-arm morbidity in breast cancer survivors submitted to breast-conserving therapy or mastectomy followed by immediate breast reconstruction. Clinics.

[19] Engel J, Kerr J, Schlesinger-Raab A, Sauer H, Hölzel D (2004). Quality of life following breast-conserving therapy or mastectomy: results of a 5-year prospective study. Breast J.

[20] EORTC Quality of Life Group. EORTC QLQ-C30 (version 3) Accessed from: https://www.eortc.org/app/uploads/sites/2/2018/08/Specimen-QLQC30-English.pdf. Accessed 03 Jan 2021.

[21] EORTC Quality of Life Group. EORTC QLQ-BR23. Accessed from: https://www.eortc.org/app/uploads/sites/2/2018/08/Specimen-BR23-English-1.1.pdf. Accessed 03 Jan 2021.

[22] Fayers P, Aaronson NK, Bjordal K, Sullivan M: EORTC QLQ–C30 scoring manual: European Organisation for Research and Treatment of Cancer; 1995.

[23] Scott NW, Fayers P, Aaronson NK, Bottomley A, de Graeff A, Groenvold M, Gundy C, Koller M, Petersen MA, Sprangers MA: EORTC QLQ-C30 reference values manual. 2008.

[24] Bantema-Joppe EJ, de Bock GH, Woltman-van Iersel M, Busz DM, Ranchor AV, Langendijk JA, Maduro JH, van den Heuvel ER (2015). The impact of age on changes in quality of life among breast cancer survivors treated with breast-conserving surgery and radiotherapy. Br J Cancer.

[25] Park B-W, Lee S, Lee AR, Lee K-H, Hwang SY (2011). Quality of life differences between younger and older breast cancer patients. J Breast Cancer.

[26] Chow R, Pulenzas N, Zhang L, Ecclestone C, Leahey A, Hamer J, DeAngelis C, Bedard G, McDonald R, Bhatia A (2016). Quality of life and symptom burden in patients with breast cancer treated with mastectomy and lumpectomy. Support Care Cancer.

[27] Browne JP, Jeevan R, Gulliver-Clarke C, Pereira J, Caddy CM, van der Meulen JH (2017). The association between complications and quality of life after mastectomy and breast reconstruction for breast cancer. Cancer.

[28] Epa T, Fernando A, Colonne S, Jayarajah U, Seneviratne S (2020). Long term post-treatment psychological outcomes in breast cancer patients from a tertiary care surgical unit in a developing country in South Asia. Br J Surg.

[29] Epa T, Fernando A, Colonne S, Jayarajah U, Seneviratne S. Long term post-treatment quality of life in breast cancer patients from a tertiary care surgical unit in a developing country in South Asia. Br J Surg. 2020;107:154–4.

[30] Jayasinghe R, Fernando A, Jayarajah U, Seneviratne S. Post Treatment Quality of Life among Sri Lankan Women with Breast Cancer. 2020. 10.21203/rs.21203.rs-108190/v108191. 10.1186/s12885-021-08055-5 PMC7988899 33757446

